# Identification of phenylpropanoid biosynthetic genes and phenylpropanoid accumulation by transcriptome analysis of *Lycium chinense*

**DOI:** 10.1186/1471-2164-14-802

**Published:** 2013-11-19

**Authors:** Shicheng Zhao, Pham Anh Tuan, Xiaohua Li, Yeon Bok Kim, HyeRan Kim, Chun Geon Park, Jingli Yang, Cheng Hao Li, Sang Un Park

**Affiliations:** Department of Crop Science, Chungnam National University, 99 Daehak-ro, Yuseong-gu, Daejeon, 305-764 Korea; Green Bio Research Center, Cabbage Genomics Assisted Breeding Supporting Center, Korea Research Institute of Bioscience and Biotechnology (KRIBB), Daejeon, 305-806 Korea; National Institute of Horticultural and Herbal Science, RDA, Bisanro 92, Eumseong, Chungbuk, 369-873 Korea; State Key Laboratory of Forest Genetics and Tree Breeding, Northeast Forestry University, 26 Hexing Road, Harbin, 150040 China

**Keywords:** Illumina sequencing, *Lycium chinense*, Phenylpropanoids, Chlorogenic acid

## Abstract

**Background:**

*Lycium chinense* is well known in traditional Chinese herbal medicine for its medicinal value and composition, which have been widely studied for decades. However, further research on *Lycium chinense* is limited due to the lack of transcriptome and genomic information.

**Results:**

The transcriptome of *L. chinense* was constructed by using an Illumina HiSeq 2000 sequencing platform. All 56,526 unigenes with an average length of 611 nt and an N50 equaling 848 nt were generated from 58,192,350 total raw reads after filtering and assembly. Unigenes were assembled by BLAST similarity searches and annotated with Gene Ontology (GO) and Kyoto Encyclopedia of Genes and Genomes (KEGG) orthology identifiers. Using these transcriptome data, the majority of genes that are associated with phenylpropanoid biosynthesis in *L. chinense* were identified. In addition, phenylpropanoid biosynthesis-related gene expression and compound content in different organs were analyzed. We found that most phenylpropanoid genes were highly expressed in the red fruits, leaves, and flowers. An important phenylpropanoid, chlorogenic acid, was also found to be extremely abundant in leaves.

**Conclusions:**

Using Illumina sequencing technology, we have identified the function of novel homologous genes that regulate metabolic pathways in *Lycium chinense*.

**Electronic supplementary material:**

The online version of this article (doi:10.1186/1471-2164-14-802) contains supplementary material, which is available to authorized users.

## Background

Plant phenylpropanoids are a group of phenylalanine-derived physiologically active secondary metabolites, such as lignins, flavonols, isoflavonoids, and anthocyanins [1]. They perform a vast array of important functions; for example, lignins reinforce specialized cell walls [2], flavonoids and isoflavonoids are involved in UV filtration [3, 4] and symbiotic nitrogen fixation [5], and anthocyanins can protect plants against damaging photo-oxidative effects and UV irradiation [6]. Phenylpropanoids also have many beneficial functions for human health including anticancer [7] and anti-inflammatory properties [8].

**Figure 1:**
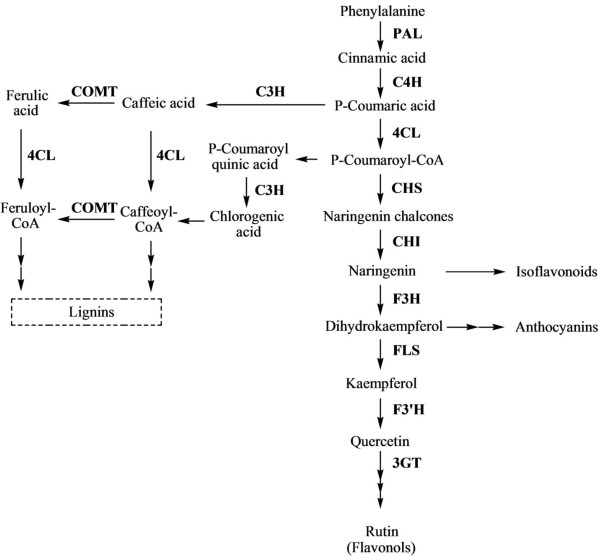
**Schematic representation of phenylpropanoid biosynthesis in** ***L. chinense*** **.** PAL, phenylalanine ammonium lyase; C4H, cinnamic acid 4-hydroxylase; 4CL, 4-coumarate-CoA ligase; CHS, chalcone synthase; CHI, chalcone isomerase; F3H, flavanone-3-hydroxylase; FLS, flavonol synthase; F3’H, flavonoid-3’-hydroxylase; 3GT, flavonoid 3-O-glucosyltransferase; C3H, p-coumarate-3- hydroxylase; and COMT, caffeate O-methyltransferase.

Phenylpropanoid biosynthesis starts with the formation of the aromatic amino acid phenylalanine. Phenylalanine ammonia lyase (PAL) catalyzes the phenylalanine into cinnamic acid. Cinnamate 4-hydroxylase (C4L) and 4-coumarate-CoA ligase (4CL) then catalyze the conversion of cinnamic acid to p-coumaroyl-CoA, which is the precursor for many phenylpropanoid products. Anthocyanins, flavonols and isoflaconoids are synthesized from p-coumaroyl-CoA through a complex phenylpropanoid pathway (Figure 1).

*Lycium chinense*, which is a member of the Solanaceae family, is a famous traditional Chinese herbal medicine that has a large variety of benefits, including anti-inflammatory effects [9], immunomodulating activity [10], anti-cancer properties [11], ability to reduce blood glucose and lipid levels [12], and anti-aging properties [13]. Researchers have isolated different secondary metabolites from *L. chinense*, such as alkaloids [14], carotenoids [15], lignans [16], and betaine [17]. Partial sequences of specific genes have also been cloned in order to evaluate gene expression during the plant’s development and to investigate the relationship of different genes in various plants [18, 19].

In recent years, next-generation sequencing (NGS) technologies such as 454 and Illumina platforms have been widely used in gene sequencing, most notably, in human genome sequencing [20, 21]. Compared with traditional gene cloning, NGS technology has high efficiency (whole-genome sequencing), fast run times (ranging from hours to days) and high accuracy [20, 22]. Among the different types of NGS technology, the Illumina Hiseq system has been widely used [23–25], owing to its high throughput, accuracy, and low costs.

The purpose of the current study was to investigate the phenylpropanoid biosynthetic pathway in *L. chinense.* Several full-length cDNAs encoding *PAL*, *C4H*, *4CL*, *CHS*, *C3H*, and *COMT*, and partial-length cDNAs encoding *CHI*, *F3H*, *FLS*, *F3’H*, and *3GT* were identified and isolated. In addition, the relationship between the transcription levels of phenylpropanoid biosynthetic genes and phenylpropanoid accumulation were analyzed in different organs of *L. chinense*. To our knowledge, this study is the first to utilize transcriptome sequencing to investigate phenylpropanoid biosynthetic correlative gene expression in different organs of *L. chinense.*

## Results and discussion

### Sequencing and sequence assembly

**Figure 2:**
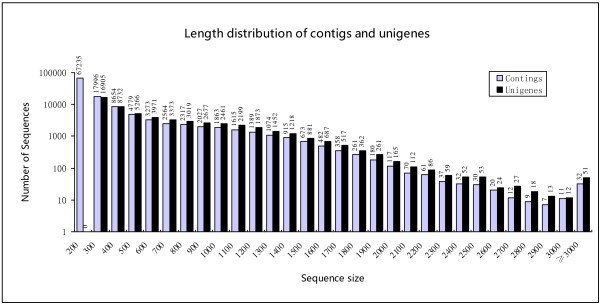
**Length distribution of contigs and unigenes in** ***L. chinense*** **.**

Whole plantlet cDNA libraries were sequenced using the Illumina HiSeq™ 2000 system. After cleaning and quality checks, we obtained more than 54.1 million clean paired-end short reads of 90 nt in length following sequencing (Table 1). The Q20 percentage (proportion of nucleotides with quality value larger than 20 in reads), N percentage, and GC percentage were 97.67%, 0.00% and 41.41%, respectively. To facilitate sequence assembly, these reads were assembled using the Trinity program, resulting in 118,093 contigs with an average contig length of 312 nt and an N50 of 511 nt, ranging from 200 nt to >3,000 nt (Table 1, Figure 2). Furthermore, Trinity was used to assemble 56,526 unigenes with a mean size of 611 nt and an N50 of 848 nt (Table 1). The unigene size distribution showed the following: 14.25% (16,823) of the unigenes were between 500 and 1000 nt in length and 79.50% (93,885) were less than 500 nt long; 7.80% (9,216) of contigs were between 1000 and 3000 nt, and 0.03% (32) were more than 3000 nt long (Figure 2).

**Table 1:** **Summary of the transcriptome of** ***L*** *.* ***chinense***

	Total number	Total nucleotides (nt)	Mean length (nt)	N50
Total raw reads	58,192,350	–	–	–
Total clean reads	54,138,216	4,872,439,440	–	–
Total Contigs	118,093	–	312	511
Total unigenes	56,526	–	611	848

### Unigene function annotation and pathways

**Figure 3:**
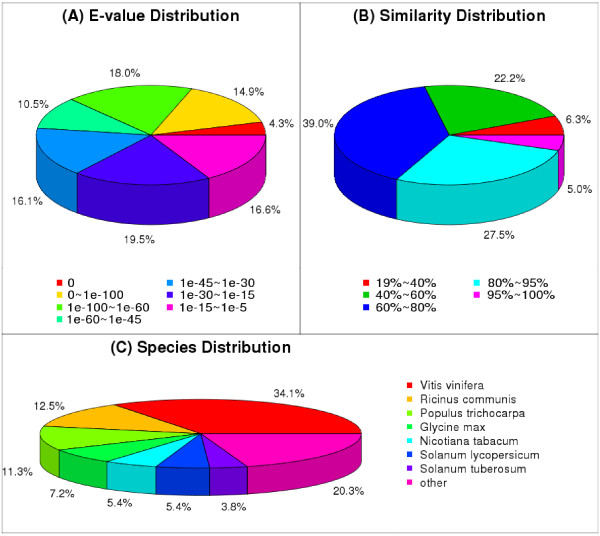
**Figure of NR classification. (A)** E-value distribution. **(B)** Similarity distribution. **(C)** Species distribution.

For annotation, the unigenes were further analyzed using BLASTX, on the National Center for Biotechnology Information (NCBI) website, against the non- redundant (nr) protein database with a cut-off E-value of 10^-5^; this resulted in the annotation of 34,684 unigenes (61.36% of all 56,526 cleaned unigenes) (Table 2). The E-value distributions of the unigenes in the Nr database showed that 37.2% of the unigenes had strong similarity (smaller than 1e-60), while the remaining 62.8% of the homologous sequences ranged from 1e-5 to 1e-60 (Figure 3A). The rates of the similarity distributions showed that 32.5% of the sequences had a similarity higher than 80%, and 67.5% of the sequences had a similarity ranging from 19% to 80% (Figure 3B). The species distributions for the best match from each sequence are shown in Figure 3C. In detail, 34.07% of the unigenes had the highest homology to genes from *Vitis vinifera*, followed by *Ricinus communis* (12.47%), *Populus trichocarpa* (11.35%), *Glycine max* (7.21%), *Nicotiana tabacum* (5.41%), *Solanum lycopersicum* (5.38%), *Solanum tuberosum* (3.77%).

**Table 2:** **Summary of annotations of the** ***L. chinense*** **unigenes**

	Number of blasted unigenes	Ratio
All unigenes	56,526	–
Unigenes blasted against NR	34,684	61.36%
Unigenes blasted against NT	38,843	68.72%
Unigenes blasted against SWISS-PROT	20,929	37.03%
Unigenes blasted against KEGG	18,596	32.90%
Unigenes blasted against COG	10,831	19.16%
Unigenes blasted against GO	26,470	46.82%
All annotated unigenes	42,022	74.34%

**Figure 4:**
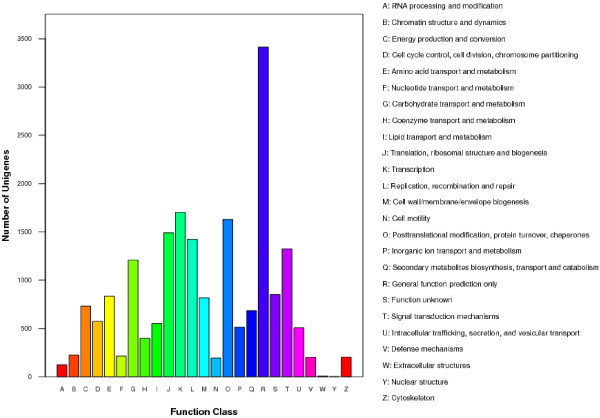
**COG function classification of all unigenes.**

**Figure 5:**
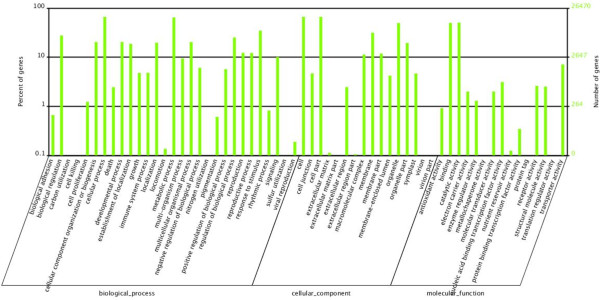
**GO annotation of all unigenes.** Annotated sequences were classified into three major categories (biological processes, cellular components, and molecular function) and 60 subgroups.

Other databases were also used to compare the unigenes, including 20,929 (37.03% of all cleaned unigenes) sequences in SWISS-PROT, 18,596 (32.90% of all cleaned unigenes) sequences in KEGG, 10,831 (19.16% of all cleaned unigenes) sequences in Clusters of Orthologous Groups (COG), and 26,470 (46.83% of all cleaned unigenes) sequences in Gene Ontology (GO) with the same identical cut-off E-value to supplement the annotations and functions. In total, 42,022 annotated transcripts were identified, representing approximately 74.34% of all cleaned unigenes (Table 2). Unigenes were compared with COGs in order to predict and classify their possible functions. The data comparison enabled the classification of 26 molecular families; the top category was “General function prediction only” (3,413 unigenes, 31.51%) (Figure 4). For GO analysis, unigenes were divided into three major categories: biological processes, cellular components, and molecular function. Among the cluster of biological processes, cellular processes and metabolic processes were the two largest groups, containing 17,530 (66.23%) and 17,089 (64.56%) unigenes respectively. In the cellular component cluster, cells, cell parts, and organelles were dominant, containing 17,574 (66.39%), 17,572 (66.38%) and 13,141 (49.64%) unigenes respectively. In the molecular function group, binding and catalytic activity were largest two sub-categories, containing 13,223 (49.95%) and 13,422 (50.71) unigenes, respectively (Figure 5). All annotated unigenes were mapped to the KEGG database to define the cellular pathways containing these unigenes (Additional file 1). A total of 18,586 unigenes were assigned to 128 pathways. The most dominant clusters were metabolic pathways (4,489 unigenes, 24.14%), followed by biosynthesis of secondary metabolites (2,459 unigenes, 13.22%), plant hormone signal transduction (1,088 unigenes, 5.85%), and plant-pathogen interaction (992 unigenes, 5.33%).

### Analysis of phenylpropanoid biosynthesis pathway genes from L. chinense unigenes

The sequences of phenylpropanoid biosynthesis pathway genes were identified in the NGS of the *L. chinense* database. They were confirmed for homology with the BLAST program and designed as *LcPAL* (719 amino acids), *LcC4H* (506 amino acids), *Lc4CL* (566 amino acids), *LcCHS* (377 amino acids), *LcCHI* (184 amino acids), *LcF3H* (218 amino acids), *LcFLS* (246 amino acids), *LcF3’H* (71 amino acids), *Lc3GT* (122 amino acids), *LcC3H* (523 amino acids), and *LcCOMT* (378 amino acids). The data provided in Table 3 show that the phenylpropanoid biosynthetic genes from *L. chinense* exhibited high identity with other orthologous genes.

**Table 3:** **Comparison of phenylpropanoid biosynthetic genes of** ***L. chinense*** **with the most orthologous genes**

Genes of ***L. chinense***	Length (amino acid)	Orthologous genes (Accession no.)	Identity (%)
*LcPAL*	719	*Petunia axillaris* PAL1 (JF793918)	85
		*Platycodon grandiflorus* PAL2 (JN392749)	78
		*Coffea arabica* PAL1 (JQ946534)	76
*LcC4H*	506	*Capsicum annuum* C4H (AF212318)	90
		*Petunia hybrida* C4H2 (HM447145)	90
		*Lithospermum erythrorhizon* C4H2 (AB055508)	82
*Lc4CL*	566	*Nicotiana tabacum* 4CL1 (U50845)	89
		*Petunia hybrida* 4CL (JN120849)	87
		*Solanum lycopersicum* 4CL1 (XM_004235822)	87
*LcCHS*	377	*Solanum lycopersicum* CHS (XM_004239850)	84
		*Petunia hybrida* CHS (X14592)	85
		*Senna alata* CHS (AF358432)	73
*LcCHI*	184	*Nicotiana tabacum* CHI1 (AB213651)	89
		*Capsicum annuum* CHI (FJ705843)	89
		*Solanum lycopersicum* CHI (XM_004238946)	91
*LcF3H*	218	*Solanum tuberosum* F3H (AY102035)	87
		*Nicotiana tabacum* F3H (AB289450)	91
		*Capsicum annuum* F3H (FJ705844)	89
*LcFLS*	246	*Solanum tuberosum* FLS (FJ770475)	91
		*Nicotiana tabacum* FLS1 (DQ435530)	91
		*Solanum lycopersicum* FLS (XM_004250281)	89
*LcF3’H*	71	*Solanum lycopersicum* F3’H(XM_004235959)	87
		*Petunia hybrida* F3’H (AF155332)	88
		*Antirrhinum majus* F3’H (DQ272592)	78
*Lc3GT*	122	*Nicotiana tabacum* 3GT (AB723686)	88
		*Petunia hybrida* 3GT (AB027454)	85
		*Capsicum annuum* 3GT (JN808443)	91
*LcC3H*	523	*Coffea arabica* C3H25 (JQ946542)	77
		*Populus trichocarpa* C3H3 (EU603301)	73
		*Trifolium pratense* C3H (GQ919201)	73
*LcCOMT*	378	*Capsicum annuum* COMT (AF212316)	84
		*Capsicum chinense* COMT (AF081214)	87
		*Catharanthus roseus* COMT1 (AY028439)	79

### Expression analysis of phenylpropanoid biosynthetic genes in different organs of L. chinense

**Figure 6:**
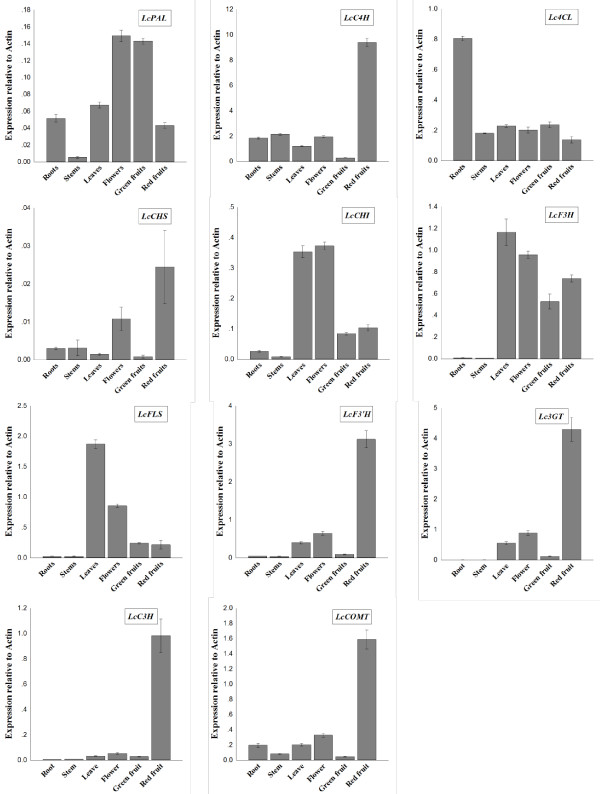
**Expression of phenylpropanoid biosynthetic genes in different organs of** ***L. chinense*** **.** Each value is the mean of three replicates, and error bars indicate SDs.

The expression of phenylpropanoid biosynthetic genes was analyzed in the roots, stems, leaves, flowers, green fruits, and red fruits of *L. chinense* by real-time PCR (Figure 6). *LcPAL*, the first enzyme in the phenylpropanoid biosynthetic pathway, was expressed at the highest levels in the flowers and green fruits, but was moderately expressed in the leaves, roots and red fruits, and only present at low levels in the stems. The expression patterns of *LcC4H*, *LcF3’H*, *Lc3GT*, *LcC3H*, and *LcCOMT* were similar, with observably higher expression in the red fruits than in the roots, stems, leaves, flowers, and green fruits. Among the phenylpropanoid biosynthetic genes of *L. chinense*, only *Lc4CL* was highly expressed in the roots, with similarly lower levels of expression in the other five organs. *LcCHS* exhibited high expression levels in the red fruits and flowers, but lower levels in the other four organs. *LcCHI*, *LcF3H* and *LcFLS* were expressed at high levels in the leaves and flowers, at intermediate levels in the green and red fruits, and at very low levels in the roots and stems.

### Analysis of phenylpropanoid content in different organs of L. chinense

**Figure 7:**
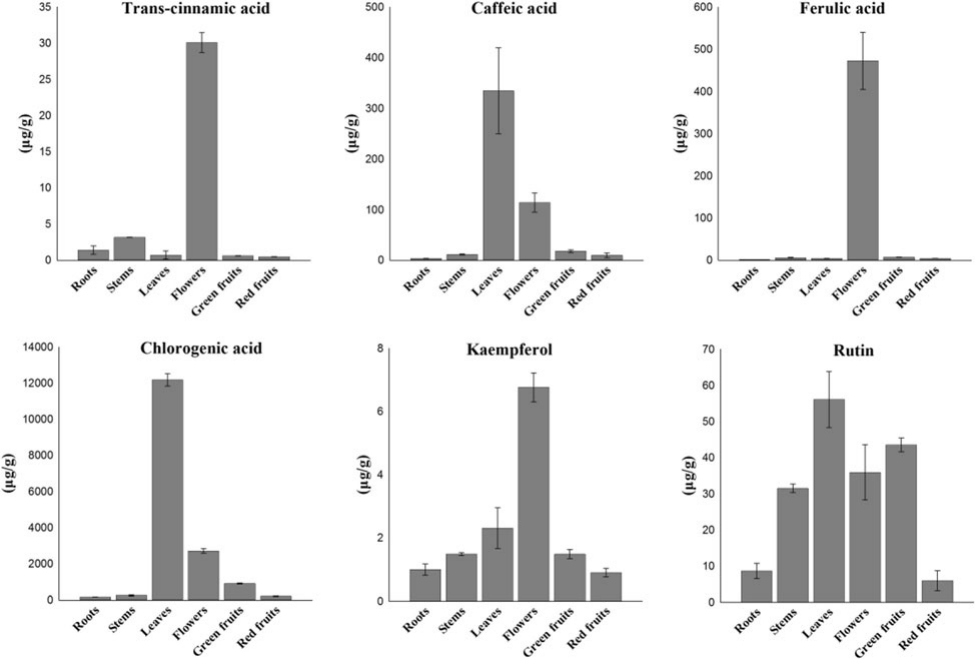
**Accumulation of phenylpropanoids in different organs of** ***L. chinense*** **.** Each value is the mean of three replicates, and error bars indicate SDs.

The same plant materials used for quantitative real-time PCR were used for the HPLC analysis of phenylpropanoid accumulation. Trans-cinnamic acid, caffeic acid, ferulic acid, chlorogenic acid, kaempferol, and rutin were measured in the different organs of *L. chinense* (Figure 7). Small amounts of trans-cinnamic acid, caffeic acid, and ferulic acid were detected in the roots, stems, green fruits, and red fruits. In flowers, all of the identified compounds were very found to be present at high levels. The leaves contained abundant amounts of chlorogenic acid (12,181.98 μg/g dry weight), caffeic acid (334.85 μg/g), and rutin (56.14 μg/g). High amounts of rutin were also found in green fruits (43.53 μg/g), flowers (35.99 μg/g), and stems (31.54 μg/g).

*LcPAL,* which carries out the first catalysis step in the phenylpropanoid biosynthesis pathway, was highly expressed in flowers in *L. chinense*, and the metabolite of this gene, cinnamic acid, was correspondingly the highest in flowers. The gene expression analysis showed that the expression level of *LcPAL* in green fruits was also high; however, the content of its metabolites in green fruits was very low. This is probably owing to the fact that *PAL*s are codified by a multigene family in plants [26, 27], suggesting the existence of other *PAL* isoforms in green fruits. In previous study of our laboratory, the expression of *C4H* in different organs was diverse between different species. In plant *Allium. sativum*, the highest expression level occurred in the root, but in plant *Agastache. rugosa*, *ArC4H* was expressed highest in flowers [28, 29]. In *L. chinense*, we found that the expression levels of *LcC4H* in red fruits was significantly higher than that that in other organs. *Lc4CL* was highly expressed in root, this result is consistent with our laboratory’s previous study in tartary buckwheat Hokkai T10 [30]. But in *L. chinense*’s roots, the expression of *Lc4CL* was significantly higher than other organs. In our laboratory’s previous study, they also found two isoform *FLS* genes in tartary buckwheat, and that two *FLS* gene expressions were different in different organs [31]. This explains why the expression of the *LcFLS* was inconsistent with that of phenylpropanoid product, kaempferol. To content of kaempferol in *L. chinense*, it’s much similar with that in buckwheat, highly accumulate in flowers than other organs. We found that the expression levels of *LcC4H*, *LcCHS*, *LcF3’H*, *Lc3GT*, *LcC3H*, and *LcCOMT* in red fruits was significantly higher than that in other plant organs but that the levels of the corresponding phenylpropanoids in red fruits was lower. It is well known that phenylpropanoid biosynthesis is a very complex process and that multiple enzyme metabolites are produced. Moreover, some enzymes exist in many isoforms. Therefore, we felt it was not appropriate to relate gene expression and metabolite production absolutely. As shown in Figure 7, the content of caffeic acid and chlorogenic acid in different organs has an essentially similar trend, where it is highest in leaves, followed by flowers; lower levels are present in other organs. In the phenylpropanoid biosynthesis pathway (Figure 1), caffeic acid and chlorogenic acid are regulated enzymes encoded by the same gene, *C3H*.

In the current study, the content of chlorogenic acid was found to be very high in leaves (12,181.98 μg/g). Chlorogenic acid is a polyphenol compound found in plants and animals and has many biological functions, including antioxidant [32], anti-stress [33], and antiviral [34] properties. Dong et al. [35] investigated the impact of selenium on the content of chlorogenic acid in leaves of *L. chinense*; the study found that the chlorogenic acid content (10,846 μg/g) in the presence of 0.01 μg/g sodium selenite was much higher than that in the control plants (78.36 μg/g). Therefore, we speculate that there were trace amounts of selenium at the experimental farm from which the *L. chinense* plants used in this study were derived. The presence of environmental elements, such as selenium, may produce certain effects on other phenylpropanoids [36]. In this stdy, we found most phenylpropanoids were accumulate in flowers and leaves, some phenylpropanoids such as kaempferol and rutin also have high accumulate in stems and green fruits,but the content of phenylpropanoids were lowest in roots and red fruits. Nowadays, most consumer purchase fresh or dry red fruits as part of diet. As a reault, we suggest consumer to use other parts of *L. chinense* to maximize the health benefits of phenylpropanoids.

## Conclusions

In this study, we developed a rapid and cost-effective method for transcriptome analysis of *Lycium chinense* using Illumina sequencing technology. Using the annotation, we found many transcripts that encode for putative genes that are involved in phenylpropanoid biosynthesis. But not all genes were found, that mostly because the sample which used for RNA sequencing was not the stage that all genes were expressed. To extensively study the genes related the biosynthesis of secondary metabolites, we have plan to use other tissues of *L.chinense* for RNA sequencing in future. We also compared gene expression in different organs. We noticed that most phenylproanoid genes were highly expressed in the leaves, flowers, and red fruits. Our compound analysis indicated that a number of phenylpropanoids were present in the leaves and flowers of *L. chinense*, such as chlorogenic acid, caffeic acid, and rutin.

## Methods

### Plant material and RNA extraction

Plant materials were collected from *Lycium chinense* cutting seedling grown outdoor at an experimental farm at Chungnam National University for 1 year (Daejeon, Korea). Whole plantlets induced from the twig segment of *L. chinen*se were used for transcriptome analysis [37]. Different organs from the mature plant, including the roots, stems, leaves, flowers, and fruits from two different stages of maturation (green fruits and red fruits), were excised. All samples were immediately frozen in liquid nitrogen and then stored at -80°C and/or freeze-dried for RNA isolation and/or high-performance liquid chromatography (HPLC) analysis. The *L. chinen*se samples were ground into powder in a mortar with liquid nitrogen, and total RNA was isolated separately using the RNeasy Plant Mini kit (Qiagen, Valencia, CA).

### Illumina sequencing

Beads with Oligo (dT) were used to isolate poly (A) mRNA after total RNA was extracted. Fragmentation buffer was then added to digest the mRNA into short fragments. Using these short fragments as templates, random hexamer primers was used to synthesize the first-strand cDNA. The second-strand cDNA was then synthesized using buffer, dNTPs, RNase H, and DNA polymerase I. Short fragments were purified with a QiaQuick PCR extraction kit and resolved with EB buffer for end reparation and addition of poly (A). Following this, the short fragments were connected with sequencing adapters and analyzed by agarose gel electrophoresis in order to select suitable fragments for amplification by PCR. The resulting cDNA library was then sequenced using an Illumina HiSeq™ 2000 system. Illumina Sequencing was performed at the Beijing Genomics Institute (BGI) genomic Center in Shenzhen, China (http://www.genomics.cn).

### Reads filtration and assembly

Image data output from the sequencer was transformed by base calling into sequence data, also called raw data or raw reads. Before the assembly, the raw reads contain filtered reads and have adapters, a proportion of unknown nucleotides larger than 5% (N ≥ 5%), duplication sequences, and low quality bases (more than 20% nucleotides with quality value ≤ 10), which negatively affect the subsequent bioinformatics analysis. Therefore, dirty raw reads were removed. The clean reads were then assembled using Trinity software (release-20120608) [38]. Trinity first combines reads with a certain length of overlap to form longer fragments, which are called contigs. The reads are then mapped back to the contigs. Finally, Trinity connects the contigs and gets sequences that cannot be extended on either end. Such sequences are defined as unigenes. Unigenes from each sample’s assembly can be used in further processes of sequence splicing and redundancy removal with sequence clustering software in order to acquire non-redundant unigenes that are as long as possible. The sequencing data from this study were deposited in the NCBI Sequence Read Archive (SRA, http://www.ncbi.nlm.nih.gov/Traces/sra/) under accession number SRR886280.

### Unigene function annotation

Information was obtained regarding functional annotation, including protein functional annotation, COG functional annotation, and GO functional annotation of unigenes. Unigene sequences were first submitted to protein databases for alignment and comparison by BLASTX algorithms with a significant threshold of E-value ≤ 10^-5^, like nr, SWISS-PROT, KEGG, and COG. In addition, unigenes were aligned by Blastn to nucleotide databases nt (E-value ≤ 10^-5^), retrieving proteins with the highest sequence similarity with the given unigenes along with their protein functional annotations. In addition, the orientation of Illumina sequences that could not be obtained directly from sequencing were derived from BLAST annotations (ftp://ftp.ncbi.nih.gov/blast/executables/blast+/LATEST/). For other sequences not involved in the BLAST search, we used the ESTScan program (version 3.0.2, http://www.ch.embnet.org/software/ESTScan2.html) to predict for “CDS” and orientation. With nr annotation, the Blast2GO program (version 2.5.0, http://blast2go.com/b2ghome) was used to classify unigenes to GO terms such as molecular function, biological processes, and cellular components [39]. After obtaining GO annotations for all unigenes, WEGO software [40] was used to perform GO function classification for all unigenes and to analyze the distribution of *L. chinen*se gene functions at the macro level. Using the KEGG pathway database and nr annotation on KEGG, we could cluster multiple unigenes to the same GO terms and the same KEGG pathway [41].

### Identifying sequences of phenylpropanoids genes

Genes in the phenylpropanoid biosynthesis pathway were identified using Illumina sequencing data. They were searched for using a functional annotation file based on the candidate gene name. Following this, each search sequence was further performed by using the BLAST program in the National Center for Biotechnology Information GenBank database (http://www.ncbi.nlm.nih.gov/BLAST).

### cDNA synthesis and real-time PCR

After total RNA of different organs were extracted, the quality and concentration of different total extracted RNA were evaluated by 1% agarose gel electrophoresis and spectrophotometric analysis, respectively. For first-strand cDNA synthesis, 1 μg of high-quality total RNA was used for reverse transcription (RT) with a ReverTra Ace-kit (Toyobo Co. Ltd., Osaka, Japan). A 20-fold dilution of 20 μL of the resulting cDNA was used as a template for quantitative real-time PCR.

Based on the sequences of *LcPAL*, *LcC4H*, *Lc4CL*, *LcCHS*, *LcCHI*, *LcF3H*, *LcFLS*, *LcF3’H*, *Lc3GT*, *LcC3H* and *LcCOMT*, real-time PCR primers were designed by the Primer 3 website (http://frodo.wi.mit.edu/primer3/) (Additional file 2). The expression of these genes was calculated by relative quantification method with rhe *L.chinense Actin* housekeeping gene, which was also isolated through NGS sequences (data not shown), as a reference. For quantification of the standard, PCR products amplified from cDNA were purified, and the concentration of the products was measured in order to calculate the number of cDNA copies. Real-time PCR reaction were performed in a 20 μL reaction mixture including 5 μL of template cDNA, 10 μL of 1 × SYBR Green Real-time PCR Master Mix (Toyobo, Osaka, Japan), 0.5 μL of each primer (10 μL) and DEPC-treated water. Thermal cycling conditions were as follows: 95°C for 5 min and 40 cycles of 95°C for 15 s, 56°C for 15 s, and 72°C for 20 s. The PCR reactions were performed on a CFX96 Real-Time system (Bio-Rad Laboratories, Hercules, CA). PCR products were analyzed with the Bio-Rad CFX Manager 2.0 software. Three replications for each sample were used for the real-time PCR analysis. Values were expressed as means ± SDs.

### High performance liquid chromatography analysis

Different organs of *L. chinense* were freeze-dried at -80°C at 48 h, and then ground into a fine powder using a mortar and pestle. Phenylpropanoids were released from the *L.chinense* samples (0.02 g) by adding 3 mL of methanol containing 0.1% ascorbic acid (w/v) at 60°C for 1 h. After centrifuging (3000 rpm) the extract, the supernatant was filtered with a 0.22 μm Acrodisc syringe filter (Pall Corp.; Port Washington, NY), and then analyzed by HPLC. The phenylpropanoids were separated on C18 column (250 × 4.6 mm, 5 μm; RStech, Daejeon, Korea) by an Agilent 1100 HPLC system (Agilent Technologies France, Massy, France) that was equipped with a photodiode array detector. The mobile phase consisted of methanol, water and 0.2% acetic acid, and the column was maintained at 30°C. The flow rate was maintained at 1.0 mL/min, the injection volume was 20 μL, and the detection wavelength was 280 nm. The concentrations of phenylpropanoid compounds were determined by using a standard curve. All samples were analyzed in triplicate. Values were expressed as means ± SDs.

## Electronic supplementary material

Additional file 1: **Pathway assignment based on KEGG.** (XLS 24 KB)

Additional file 2: **Primers used for real-time PCR.** (DOC 32 KB)
