# Supplementary material for: Identification of phenylpropanoid biosynthetic genes and phenylpropanoid accumulation by transcriptome analysis of Lycium chinense
Source: BMC Genomics. 2013 Nov 19;14(1):802. doi: 10.1186/1471-2164-14-802 (PMC4046672; doi:10.1186/1471-2164-14-802)
Supplement: Supplementary file 2 — Additional file 2: Primers used for real-time PCR. (DOC 32 KB) [file 12864_2013_5533_MOESM2_ESM.doc]

Additional file 2 - Primers used for real-time PCR.

|  |  |  |
| --- | --- | --- |
| Gene | Forward (5′ to 3′) | Reverse (5′ to 3′) |
| *LcPAL* | AACACAACCAGGATGTCAACTCATT | GAACAACACTTTCTTGGCTGCTAAA |
| *LcC4H* | GGCCTTTCTTGAGAGGTTATTTGAA | GCAAGCTTCTTTCTTTCATCAACAA |
| *Lc4CL* | AGTGAGGATGTGTTGATGTGTGTGT | TTTTTGCATAATCAATATCGCTGCT |
| *LcCHS* | ATTGTCACCTCGCGTTACACCT | ATATTCCTCGTGGCCCGTAACT |
| *LcCHI* | AACCTACGATGATGCAGAAAGTCAG | ATGACCCAAGTGGTGATTGAGTAAA |
| *LcF3H* | TTAGAGAAGGAGGCCTTAACCAATG | TGTGTCTTTTCAGCCCAAGAGTAAG |
| *LcFLS* | CAGGCTCACAGAGTATTGAAGGGTA | GAGTATTCCTCATTTGCTTCCCTGT |
| *LcF3’H* | CAATTGCCAGAGAAACTAAACATGG | GTATACTTGGGCTTCTAAGCGAGGA |
| *Lc3GT* | TTTCAATGAGGAAAAAGGGAAGG | ACAATCAAGTGGCCTTGTGACAT |
| *LcC3H* | CATTTCGTTGATGCCTTGCTTAC | TTTGTTGCACTCTTGGGTTCCTA |
| *LcCOMT* | AGTACCATGGCACAGATCCAAGA | TAATGGAAGGATGCTTGGAGACA |
